# The Relationship Between Working Alliance and Symptom Improvement in Cognitive Therapy for Posttraumatic Stress Disorder

**DOI:** 10.3389/fpsyt.2021.602648

**Published:** 2021-04-16

**Authors:** Esther T. Beierl, Hannah Murray, Milan Wiedemann, Emma Warnock-Parkes, Jennifer Wild, Richard Stott, Nick Grey, David M. Clark, Anke Ehlers

**Affiliations:** ^1^Department of Experimental Psychology, University of Oxford, Oxford, United Kingdom; ^2^Oxford Health NHS Foundation Trust, Oxford, United Kingdom; ^3^Department of Psychology, King's College London, London, United Kingdom; ^4^South London and Maudsley NHS Foundation Trust, London, United Kingdom; ^5^Psychology and Psychological Therapies, Sussex Partnership NHS Foundation Trust, Worthing, United Kingdom

**Keywords:** posttraumatic stress disorder, cognitive therapy, working alliance, cross-lagged associations, treatment outcome

## Abstract

**Background:** Working alliance has been shown to predict outcome of psychological treatments in multiple studies. Conversely, changes in outcome scores have also been found to predict working alliance ratings.

**Objective:** To assess the temporal relationships between working alliance and outcome in 230 patients receiving trauma-focused cognitive behavioral treatment for posttraumatic stress disorder (PTSD).

**Methods:** Ratings of working alliance were made by both the patient and therapist after sessions 1, 3, and 5 of a course of Cognitive Therapy for PTSD (CT-PTSD). Autoregressive, cross-lagged panel models were used to examine whether working alliance predicted PTSD symptom severity at the next assessment point and vice versa. Linear regressions tested the relationship between alliance and treatment outcome.

**Results:** Both patients' and therapists' working alliance ratings after session 1 predicted PTSD symptom scores at the end of treatment, controlling for baseline scores. At each assessment point, higher therapist working alliance was associated with lower PTSD symptoms. Crossed-lagged associations were found for therapist-rated alliance, but not for patient-rated alliance: higher therapists' alliance ratings predicted lower PTSD symptom scores at the next assessment point. Similarly, lower PTSD symptoms predicted higher therapist working alliance ratings at the next assessment point. Ruminative thinking was negatively related to therapists' alliance ratings.

**Conclusions:** Working alliance at the start of treatment predicted treatment outcome in patients receiving CT-PTSD and may be an important factor in setting the necessary conditions for effective treatment. For therapists, there was a reciprocal relationship between working alliance and PTSD symptom change in their patients during treatment, suggesting that their alliance ratings predicted symptom change, but were also influenced by patients' symptom change.

## Introduction

The working alliance, an important aspect of the therapeutic relationship, defined broadly as the “collaborative and affective bond between the therapist and patient” (1), has long been considered an essential component in the successful delivery of psychological therapy (2). Research findings have generally supported this assumption, with moderate but consistent associations found between alliance ratings and treatment outcome across different therapeutic approaches and disorders (1, 3). However, effect sizes are often in the small to moderate range; Horvath et al. (4) estimated an effect of *r* = 0.28 based on 190 alliance-outcome relationships reported in 201 studies. This is similar to results reported in previous meta-analyses with estimates of *r* = 0.26 [24 studies (3)] and *r* = 0.22 [79 studies (1)]. These associations are found whether the alliance rating is made by the patient, therapist or an observer. Some, but not all, studies have found that patients' alliance ratings are better predictors of outcome than therapists' or observers' (1, 3). Similarly, patients' ratings tend to be more consistent across therapy sessions than therapists' (1), suggesting that patients view the alliance as more stable. This finding requires replication, as few studies include ratings taken from both patient and therapist at multiple time points.

Studies investigating the predictive power of the working alliance have found differing effects depending on the time point at which the alliance is recorded. DeRubeis and Feeley (5) found that observer-rated working alliance measured in an early session of treatment for depression did not predict subsequent symptom change. However, symptom reduction during treatment predicted alliance later in therapy, raising the intriguing possibility that it is improvement in therapy which predicts how positively the alliance is viewed, rather than the other way around. Many studies have averaged alliance ratings taken across therapy (4) obscuring the temporal order, and therefore the causation relationship between alliance and outcome.

Studies which have investigated the temporal relationship between alliance ratings and outcome have produced mixed findings, with some reporting a relationship between alliance and treatment outcome (6–9), while others did not find evidence for a significant association (10, 11). The possibility that symptom change predicts later alliance ratings has also been replicated in several studies (7, 9, 12). A reciprocal relationship, whereby alliance is found to predict symptom improvement and vice versa has also been demonstrated (13, 14).

A number of studies have shown that a good working alliance predicts better treatment outcome in patients with PTSD [see (15) for a review]. However, most of these studies have used a pooled or single point measure of working alliance and have not examined the relationship in the opposite direction (i.e., symptom change influencing alliance). This study aims to assess both directions of the relationship by taking ratings of working alliance at three time points (after sessions 1, 3, and 5) within the treatment arc. This allows a more rigorous examination of the longitudinal relationships between the working alliance and treatment outcome in the early phase of treatment where the greatest changes in symptoms are observed (Ehlers et al., 2021)^1^. Ratings taken by both patients and therapists will be analyzed in a cohort of patients being treated for posttraumatic stress disorder (PTSD) using Cognitive Therapy for PTSD [CT-PTSD (16)], which is based on (17) cognitive model of PTSD. Working alliance has only been assessed in CT-PTSD in one previous study, where Brady et al. (18) compared high and low treatment responders on an observer-rated version of the Working Alliance Inventory (WAI) and found that the alliance/agreement component of the scale (comprising items on the task and goals of therapy), but not the relationship (or bond) component predicted better outcome. Brady et al. (18) also found that a perseverative thinking style (ruminative thinking) was related to lower working alliance and poorer outcomes. This study will explore these findings with a larger cohort and with patient and therapist ratings, including analysis of sub-scales of the WAI. Given the importance of ruminative thinking identified in Brady et al.'s study, we will also explore its association with working alliance and outcome, by analyzing whether rumination correlates with ratings of working alliance.

There may be reason to argue that the working alliance is particularly important in treatment for PTSD [e.g., (15)]. CT-PTSD, and most other evidence-based treatments, are trauma-focused, relying on the disclosure of intensely personal and painful experiences. Furthermore, avoidance of reminders of the trauma, as well as cognitive and emotional avoidance, are symptoms of PTSD, so a strong therapeutic alliance is needed to encourage patients to overcome their avoidance of talking about or thinking about their trauma. Lastly, many people with PTSD have experiences of interpersonal trauma, interpersonal difficulties and poor trauma-related social support, which have been shown to impede the development of a trusting alliance (19, 20). In this study, the effect on working alliance of interpersonal vs. non-interpersonal traumas will be assessed, and entered as a potential moderator in the relationship between working alliance and outcome.

**Figure 1:**
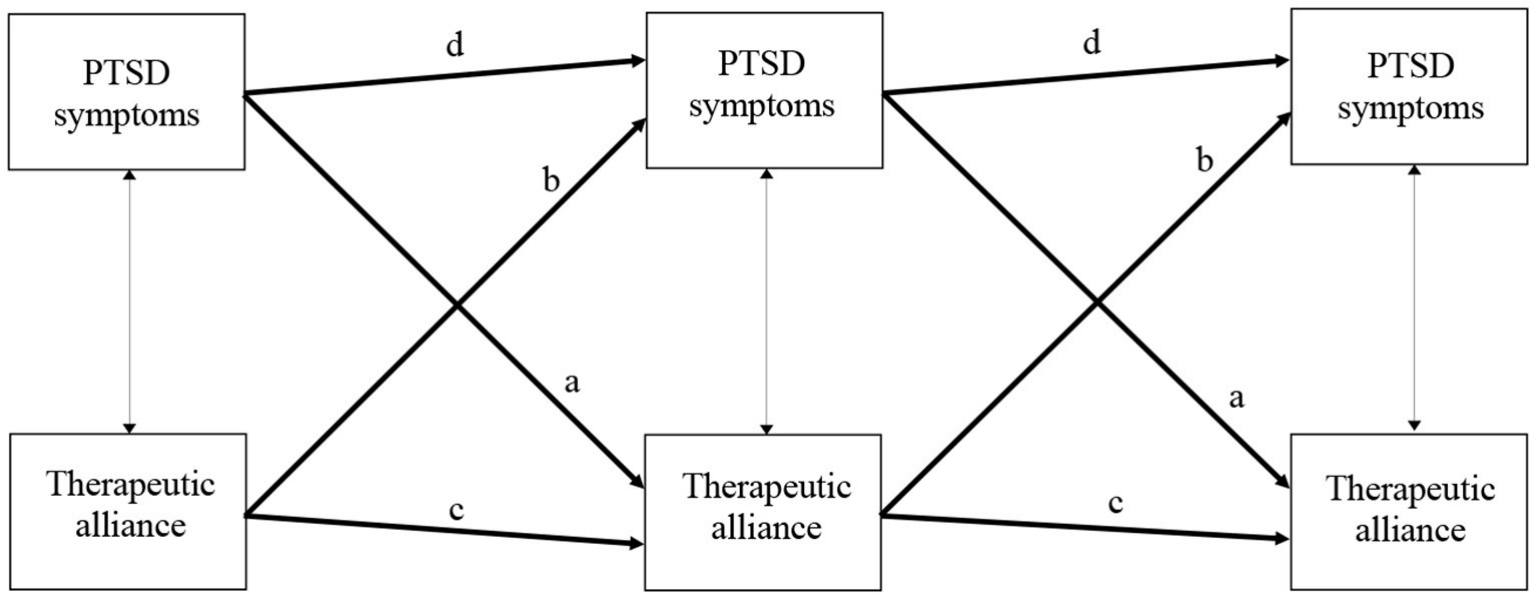
Schematic figure of the hypothesized autoregressive, cross-lagged models. Thick paths with arrows in one direction, such as **a** (PDS_t_ → WAI_t+1_; t refers to the respective treatment session and t+1 to 2 sessions later) and **b** (WAI_t_ → PDS_t+1_) indicate cross-lagged effects, paths **c** (WAI_t_ → WAI_t+1_) and **d** (PDS_t_ → PDS_t+1_), indicate autoregressive effects; thin paths with arrows in both directions represent correlations at the same session (PDS_t_ ↔ WAI_t_).

The study investigated three questions:

1. **Prediction of treatment outcome**: In line with previous research, we predicted that higher working alliance rated by patients and therapists at the end of session 1 of CT-PTSD would predict better treatment outcome, measured by PTSD symptom severity at the end of treatment, controlled for baseline PTSD severity.
2. **Does working alliance drive symptom improvement during treatment or vice versa:** As previous research has yielded inconsistent results about the direction of changes in symptoms and working alliance, we investigated whether working alliance predicts symptom improvement a later session and/or vice versa (see Figure 1).
3. **Relationship of alliance with ruminative thinking**: In addition, we explored the relationship between patients' ruminative thinking style and patient and therapist ratings of working alliance, building on Brady et al.'s (18) results that ruminative thinking is associated with lower agreement/confidence, a component of alliance.

## Methods

### Participants and Procedure

The current study is a secondary analysis of an effectiveness study of a cohort of 343 consecutive patients treated in routine clinical care (Ehlers et al., 2021)^1^ with CT-PTSD (16). Patients had experienced a range of traumas, including various forms of interpersonal violence, accidents, and death of others.

Treatment was delivered by clinical psychologists, trainee clinical psychologists, and CBT therapists and trainees with other professional backgrounds (i.e., psychiatry, nursing) with a range of clinical experience. Patients completed PTSD symptom measures weekly before every treatment session (assessing their symptoms over the preceding week) and patients and therapists both completed working alliance measures at the end of sessions 1, 3, and 5. The measure was given to the patient by a research assistant, and not seen by the therapist.

Working alliance scores and the corresponding PTSD symptom severity for the week following sessions 1, 3, and 5 were used for the analysis of the bidirectional relationships, and the interval between assessments was thus two treatment sessions. Data were available for 230 patients for whom at least one patient or one therapist alliance measure and one PTSD symptom measure was available at the respective sessions. Demographics for the study sample of *N* = 230 are presented in Table 1 and descriptive statistics are presented in Table 2.

**Table 1:** Patient characteristics (*N* = 230).

**Variable**	**n**	**%**	**M *(SD)***
Age (in years)	230		37.77 (11.63)
Months since traumatic event	228		52.38 (78.88)
Gender			
Male	100	43.5	
Female	130	56.5	
Ethnicity			
White	146	63.0	
Ethnic minority	84	37.0	
Relationship			
Married/Cohabiting	83	36.1	
Divorced/Separated/Widowed	35	15.2	
Never married	104	45.2	
No information	8	3.5	
Education			
University	67	29.1	
A-levels	29	12.6	
GCSE	49	21.3	
Professional qualification	20	8.7	
No formal qualification	19	8.3	
No information	46	20.0	
Employment			
Employed/Self-employed	104	45.2	
Sick leave	10	4.3	
Disability/Retired	14	6.1	
Unemployed	73	31.7	
Other	5	2.2	
Student	10	4.3	
No information	14	6.1	
Type of main traumatic event			
Interpersonal violence	150	65.2	
Accident or disaster	44	19.1	
Death or harm to others	24	10.4	
Other	12	5.2	

*n, number of available responses for each variable; %, percentage of study sample*.

**Table 2:** Descriptive statistics of PTSD symptoms and working alliance (*N* = 230).

**Assessment**	**Measure**	***M (SD)***	***n***
Initial	PDS	33.78 (9.66)	230
After session 1	PDS	30.10 (11.22)	210
	${\text{WAI}}_{\text{Patient}{\text{s}}^{\text{′}}\text{rating}}$	70.89 (11.28)	152
	${\text{WAI}}_{\text{Therapist}{\text{s}}^{\text{′}}\text{rating}}$	65.06 (11.21)	179
After session 3	PDS	26.40 (12.20)	193
	${\text{WAI}}_{\text{Patient}{\text{s}}^{\text{′}}\text{rating}}$	73.62 (9.01)	130
	${\text{WAI}}_{\text{Therapist}{\text{s}}^{\text{′}}\text{rating}}$	67.06 (12.25)	150
After session 5	PDS	21.52 (12.73)	179
	${\text{WAI}}_{\text{Patient}{\text{s}}^{\text{′}}\text{rating}}$	75.12 (9.68)	105
	${\text{WAI}}_{\text{Therapist}{\text{s}}^{\text{′}}\text{rating}}$	68.88 (11.21)	132
Final session	PDS	15.60 (14.12)	230

*Sum scores for all measures are reported; 17 PDS items rated on a 4-point Likert scale ranging from 0 to 3; 12 WAI items rated on a 7-point Likert Scale ranging from 1 to 7; n = number of available responses for each variable*.

Posttraumatic stress disorder symptom data at their final treatment session and at baseline was available for all patients. Exploration of any possible patterns of missing data for the other measures is reported in the preliminary analyses. Patients received on average *M* = 9.95 (*SD* = 4.57) treatment sessions in total.

### Measures

#### PTSD Symptom Severity

Patients completed the Posttraumatic Diagnostic Scale [PDS (21)], which assesses the severity of the 17 PTSD symptoms specified in the Diagnostic and Statistical Manual of Mental Disorders, Fourth edition [DSM-IV (22)]. Patients rated the extent to which they were bothered by each of the 17 symptoms during the last week (4-point Likert scale) before each treatment session. Cronbach's α at session 1 was 0.88.

#### Working Alliance

Therapeutic working alliance was assessed by the patients and therapists using the short version (23) of the WAI at the end of sessions 1, 3, and 5. The original version of the WAI was developed by Horvath and Greenberg (24) according to Bordin's (25) three components of alliance (tasks, goals, and bond). The short version consists of 12 items (7-point Likert scale), Cronbach's α at session 1 was 0.95 for the patient ratings and 0.96 for the therapist ratings. For the prediction of treatment outcome, sub-scores of the WAI (Task, Goal, Bond) were also calculated to aid interpretation.

#### Ruminative Thinking

Ruminative thinking was measured at session 1 with the 6-item rumination subscale of the Response to Intrusions Scale (26, 27). Cronbach's α for this subscale was 0.86.

### Treatment

Cognitive Therapy for PTSD (CT-PTSD) aims to reduce the patient's sense of current threat by changing problematic meanings of the trauma and its consequences, elaborating and updating the memories of the trauma with information that gives them a less threatening meaning at present, discriminating triggers of intrusive memories, and changing behaviors and cognitive processes that maintain PTSD, such as rumination and safety behaviors.

Core interventions in CT-PTSD are: the collaborative development of an individualized case formulation; reclaiming/rebuilding your life assignments to address the clients' perceived permanent change after trauma by re-engagement with activities and relationships; changing problematic appraisals of the traumas and their sequelae via information, guided discovery and behavioral experiments; updating trauma memories by elaborating and updating the worst moments of the memory; discrimination training with triggers of reexperiencing; a site visit (returning to the scene of the trauma); dropping unhelpful behaviors and cognitive processes; a blueprint summarizing what the client has learned in treatment and planning for any setbacks. Throughout treatment, the work on appraisals is closely interwoven with memory work and is tailored to the case formulation. The specific cognitive therapy techniques depend on the client's pattern of emotions and underlying cognitive themes. For further details of treatment procedures and measures see https://oxcadatresources.com.

### Statistical Analysis

Preliminary analyses investigated any effects of PTSD symptom severity or degree of working alliance on the occurrence of missing data (coded as “1”) vs. not missing (coded as “0”) using logistic regressions; unstandardized parameter estimates are reported for these analyses. Welch tests were used to investigate any potential differences between patients who experienced interpersonal (coded as “−0.5”) compared to non-interpersonal traumas (coded as “0.5”) with regards to therapeutic alliance at the beginning of treatment and PTSD symptom severity at baseline and at the end of treatment. Moderation and simple slope analyses using multiple linear regressions investigated any effects between trauma type and alliance ratings on treatment outcome, controlled for baseline severity.

The first research question (prediction of treatment outcome by initial working alliance, controlled for baseline PTSD symptom severity) was tested using multiple linear regressions and we report unstandardized and standardized coefficients.

To investigate the second research question (whether working alliance drives symptom improvement during treatment or vice versa), autoregressive, cross-lagged panel models (28) were specified. As shown in Figure 1, these models tested effects of time for each of the variables (i.e., symptom improvements over time) and any causal effects between both variables (i.e., if working alliance drives improvement in symptoms, we would observe effects of the WAI on symptom scores at two sessions later, i.e., WAI at session 1 on symptoms at session 3, and from WAI in session 3 on symptoms at session 5, paths **b** in Figure 1; and vice versa if symptom change drives alliance change, paths **a** in Figure 1). Autoregressions (paths **c** and **d**) and cross-lag effects across sessions (paths **a** and **b**), and correlations within the same sessions were each set to be equal and freely estimated. In addition to reporting standardized (β) parameter estimates for the main research questions, unstandardized parameter estimates (b) are reported for these panel models (see Table 3). Model fit was evaluated based on the χ^2^-test statistic (29, 30) and the fit indices CFI (31), RMSEA (32), and SRMR (33). We set the criterion that at least one patient alliance score and one PTSD symptom score at the relevant sessions (either after session 1, 3, or 5) should be available for a patient to be included in the respective panel analysis (*n* = 185 patients). Similarly, at least one therapist alliance rating and one PTSD symptom score had to be available at the relevant sessions for a patient to be included in the panel model investigating therapist alliance (*n* = 213 patients). In order to include all patients within those two sub-samples (symptom or alliance data only available at one or two of the three respective sessions) into the respective panel analyses, Robust Maximum Likelihood estimation (34) was used together with Full Information Maximum Likelihood (35).

**Table 3:** Autoregressive and cross-lagged effects.

**Model**	**Effects**	***b***	***SE***	**β**	***p***
Patients' alliance^a^	(a) PDS_t_ → WAI_t+1_	−0.05	0.03	−0.05	0.173
	(b) WAI_t_ → PDS_t+1_	−0.06	0.04	−0.06	0.142
	(c) WAI_t_ → WAI_t+1_	0.72	0.04	0.79	<0.001
	(d) PDS_t_ → PDS_t+1_	0.82	0.06	0.76	<0.001
Therapists' alliance^b^	(a) PDS_t_ → WAI_t+1_	−0.12	0.04	−0.12	0.001
	(b) WAI_t_ → PDS_t+1_	−0.14	0.04	−0.13	0.001
	(c) WAI_t_ → WAI_t+1_	0.76	0.04	0.75	<0.001
	(d) PDS_t_ → PDS_t+1_	0.84	0.05	0.77	<0.001

*(a) and (b) indicate cross-lagged effects; (c) and (d) indicate autoregressive effects (see Figure 1)*.

a * n = 185*;

b * n = 213*.

The third research question (association between working alliance and ruminative thinking) was assessed with Pearson correlations (*r*).

Data were analyzed using *RStudio* (36) and the packages *lavaan* (37), *psych* (38), *sjmisc* (39), *skimr* (40), *emmeans* (41), and the *tidyverse* set of packages (42). R code for data analysis can be accessed at ETB's Open Science Framework repository (https://osf.io/4dqyx/).

## Results

### Preliminary Analyses

#### Missing Data

Whether PTSD symptom data after session 5 were missing or not did not depend on: PTSD symptom severity after session 1, *b* = −0.01, *SE* = 0.01, *b*_*p*_ = −0.16, *p* = 0.352; the degree of patients' alliance after session 1, *b* = −0.01, *SE* = 0.02, *b*_*p*_ = 0.06, *p* = 0.779; or therapists' alliance after session 1, *b* = −0.03, *SE* = 0.02, *b*_*p*_ = −0.31, *p* = 0.091.

#### Trauma Type

Patients who experienced interpersonal traumas rated their therapeutic alliance after session 1 lower than patients who experienced other types of trauma, *t*_(130.04)_ = −2.80, *p* = 0.006, whereas there was no significant difference for therapist ratings, *t*_(154.46)_ = −1.74, *p* = 0.084. Patients who experienced interpersonal compared to non-interpersonal traumas did not differ in their PTSD symptom severity at baseline, *t*_(170.74)_ = −1.17, *p* = 0.243, or at the end of treatment, *t*_(181.70)_ = 1.95, *p* = 0.053.

Trauma type (interpersonal vs. non-interpersonal) did not significantly moderate any influence of patients' alliance ratings after session 1 on PTSD symptom severity at the end of treatment, controlled for baseline PTSD symptom severity, *b* = 0.37, *SE* = 0.21, β = −0.29, *p* = 0.077, *R*^2^_adj_ = 0.15. However, a simple slope analysis revealed that, for patients who experienced interpersonal traumas, patients' therapeutic alliance after session 1 had a significant effect on reduction of PTSD symptom severity at the end of treatment, controlled for baseline severity, *b* = −0.31, 95% CI [−0.51, −0.11]. This relationship was not significantly different from zero for patients who experienced non-interpersonal traumas, *b* = −0.06, 95% CI [−0.29, 0.42].

Trauma type (interpersonal vs. non-interpersonal) also did not significantly moderate any effect of therapist' alliance ratings after session 1 on PTSD symptom severity at the end of treatment, controlled for baseline PTSD symptom severity, therapists' WAI: *b* = −0.13, *SE* = 0.17, β = 0.10, *p* = 0.460, *R*^2^_adj_ = 0.27. For both patients with interpersonal and non-interpersonal traumas, the relationship between therapists' working alliance after session 1 had a significant effect on treatment outcome, controlled for baseline severity, interpersonal trauma: *b* = −0.32, 95% CI [−0.51, 0.14]; non-interpersonal trauma: *b* = −0.45, 95% CI [−0.74, 0.16].

### Analyses of the Main Research Questions

#### Question 1: Prediction of Treatment Outcome by Early Working Alliance

Both higher patient-reported and therapist-reported working alliance after the first treatment session predicted better outcome, i.e., lower PTSD symptom severity at the final treatment session (controlled for symptom severity at baseline); patients' WAI: *b* = −0.23, *SE* = 0.09, β = −0.19, *p* = 0.008, *R*^2^_adj_ = 0.13; therapists' WAI : *b* = −0.36, *SE* = 0.08, β = −0.29, *p* < 0.001, *R*^2^_adj_ = 0.28. The results were the same if the three WAI sub-scales were considered independently (patients: Task sub-scale *p* = 0.018, Goal sub-scale *p* = 0.004, Bond sub-scale *p* = 0.018; therapists: Task *p* < 0.001, Goal *p* < 0.001, Bond *p* < 0.001).

#### Question 2: Prediction of PTSD Symptom Severity by Prior Working Alliance and Prediction of Working Alliance by Prior PTSD Symptom Severity

##### Fit Measures of the Autoregressive, Cross-Lagged Models

Both cross-lagged, autoregressive panel models for the patients' and therapists' alliance ratings fit the data well; model for patients' WAI: $\chi_{\left(10\right)}^{2}$ = 12.61, *p* = 0.247, CFI = 0.99, RMSEA [95% CI] = 0.04 [0.00, 0.10], SRMR = 0.05; model for therapists' WAI: $\chi_{\left(10\right)}^{2}$ = 22.16, *p* = 0.014, CFI = 0.97, RMSEA [95% CI] = 0.08 [0.04, 0.13], SRMR = 0.06. In the patients' alliance model, 64% of variance was explained in PTSD symptom severity and 60% of variance in patient-reported working alliance after session 5. In the therapists' alliance model, 70% of variance was explained in PTSD symptom severity and 67% of variance in therapist-reported working alliance after session 5.

##### Parameter Estimates of the Autoregressive, Cross-Lagged Models

***Patient-reported working alliance and PTSD symptom severity*.** A higher working alliance reported by patients (see Table 3 and Figure 1) was not associated with lower PTSD symptom severity at the same session, *r* = −0.08, *p* = 0.122. Higher alliance scores after sessions 1 or 3 predicted higher alliance at the next assessment (i.e., alliance ratings after the session 1 predicted higher alliance ratings after the session 3 and alliance ratings after the session 3 predicted higher alliance ratings after the session 5; paths **c** in Figure 1), β = 0.79, *p* < 0.001. Lower PTSD symptom severity in the week after sessions 1 or 3 predicted lower PTSD symptom severity at the next assessment (i.e., PTSD symptom severity after the session 1 predicted PTSD symptom severity after the session 3 and PTSD symptom severity after the session 3 predicted PTSD symptom severity after the session 5; paths **d** in Figure 1), β = 0.76, *p* < 0.001. Thus, preceding levels of patient-reported therapeutic alliance predicted subsequent levels of patients' alliance and preceding levels of PTSD symptom severity predicted subsequent levels of symptom severity.

Taking into account these autoregressive coefficients, patients' self-reported PTSD symptom severity in the week after sessions 1 or 3 did not significantly predict a higher patient-reported alliance at the next assessment (i.e., PTSD symptom severity after the session 1 did not predict alliance after the session 3 and PTSD symptom severity after the session 3 did not predict alliance after the session 5; paths **a** in Figure 1), β = −0.05, *p* = 0.173. A higher patient-reported alliance after sessions 1 or 3 also did not predict lower PTSD symptom severity at the next assessment (i.e., alliance after the session 1 did not predict PTSD symptom severity after the session 3 and alliance after the session 3 did not predict symptom severity after the session 5; paths **b** in Figure 1), β = −0.06, *p* = 0.142. Thus, preceding levels of patient-reported therapeutic alliance did neither drive subsequent improvement in PTSD symptom severity, nor vice versa.

***Therapist-reported working alliance and PTSD symptom severity*.** A higher working alliance reported by therapists (see Table 3 and Figure 1) was associated with lower PTSD symptom severity after the same session, *r* = −0.16, *p* < 0.001. Higher therapist-reported alliance after sessions 1 or 3 predicted higher therapist-reported alliance at the subsequent assessment (i.e., after the session 3 or 5; paths **c** in Figure 1), β = 0.75, *p* < 0.001, and lower PTSD symptom severity in the week after session 1 or 3 predicted lower PTSD symptom severity at the successive assessment (i.e., after the session 3 or 5; paths **d** in Figure 1), β = 0.77, *p* < 0.001. Thus, similar to the results from the auto-regressions in the patients' alliance model, preceding levels of therapist-reported alliance predicted subsequent levels of therapist-reported alliance and preceding levels of PTSD symptom severity predicted subsequent levels of symptom severity.

Taking into account these auto-regressions, lower PTSD symptom severity in the week after session 1 or 3 significantly predicted higher therapist-reported working alliance at the subsequent assessment (i.e., after the session 3 or 5; paths **a** in Figure 1), β = −0.12, *p* = 0.001, and higher therapist-reported alliance after session 1 or 3 predicted significantly lower PTSD symptoms at the subsequent assessment (i.e., after the session 3 or 5; paths **b** in Figure 1), β = −0.13, *p* = 0.001. Thus, unlike to the results from the cross-lagged parameters in the patients' alliance model, preceding levels of therapist-reported alliance did drive subsequent PTSD symptom improvement and vice versa.

#### Question 3: Relationships With Ruminative Thinking

Therapist alliance ratings in the first session showed a negative relationship with patients' ruminative thinking about the trauma in the same session, *r* = −0.19, *p* = 0.015, whereas patient alliance ratings showed a non-significant positive relationship with rumination, *r* = 0.13, *p* = 0.131.

## Discussion

This study aimed to assess whether higher working alliance predicted better treatment outcomes in patients receiving CT-PTSD. Higher working alliance at the start of treatment, as rated by both patients and therapists after session 1, was associated with greater symptom improvement, measured by symptom scores at the end of treatment, controlled for baseline scores. This extends the earlier findings of Brady et al. (18), who found that patients reporting a stronger working alliance were more likely to respond well to CT-PTSD, and replicates the findings of numerous other studies which have found a positive association between working alliance and therapy outcome, including in PTSD treatment (15). Although the effect sizes in our study were of small to medium size, they are in line with those of other studies in a range of different disorders (1, 3, 15). These results support the importance of establishing a good working relationship with patients in trauma-focused psychological therapies for PTSD, which is associated with treatment outcomes, although other processes such as reduction of negative appraisals also play a role (43). Higher ratings in the total score and all the three subscales Bond, Goal, and Task were predictive of better outcomes, suggesting that a positive relationship and agreement on mutual goals as well as agreement on concrete steps to be taken in therapy may be important in facilitating change. The alliance ratings were consistently high for both patients and therapists. The collaborative therapeutic style of CT-PTSD may have facilitated a positive working alliance.

Secondly, we aimed to find whether working alliance led to improved symptom scores or vice versa. The results from autoregressive, cross-lagged panel models in this study provided support for a bidirectional relationship between the patients' symptom improvements and working alliance rated by therapists during treatment. A measure of working alliance completed by therapists after sessions 1 and 3 of treatment predicted subsequent symptom severity (i.e., after session 3 and 5; see Figure 1), with a better alliance predicting lower symptoms scores, taking into account the preceding symptom scores. During treatment, therapist-rated alliance after session 3 and 5 was predicted by symptom scores at the preceding time point (i.e., after session 1 and 3; see Figure 1), as well as by preceding alliance ratings. This fits with other studies suggesting a reciprocal relationship between alliance and outcome (13, 14); a positive alliance leads to better therapy outcomes, and better outcomes encourage therapists to view the alliance more positively. The reciprocal relationship found for working alliance contrasts with studies showing a unidirectional relationship between changes in negative cognitions about the trauma and symptom change in the treatment of PTSD. Cognitive change preceded symptom change in studies of CT-PTSD (43) and other trauma-focused cognitive behavioral treatments (44), and a reverse relationship was found in only a small minority of studies. Taken together, these findings suggest that cognitive change drives symptom change, but a good working alliance both facilitates, and is a result of, symptom change.

However, despite the overall relationship between patient-rated working alliance at the session 1 and improvement of PTSD symptoms during therapy, no significant cross-lagged associations between patients' alliance and symptoms were found in the early sessions of therapy when taking into account the significant effects of preceding symptom scores on subsequent symptom levels, and preceding alliance scores on subsequent alliance levels. Preceding levels of patients' alliance (i.e., after sessions 1 or 3; see Figure 1) did neither predict subsequent levels of PTSD symptom severity (i.e., after session 3 or 5; see Figure 1), nor vice versa (PTSD symptom scores in the week after session 1 or 3 did not predict the working alliance at the subsequent assessment, i.e., after session 3 or 5; see Figure 1), controlled for the respective auto-correlations of symptoms scores and alliance scores over time. Thus, the results for patient-rated alliance were mixed, which is in line with the literature. Some studies have shown that working alliance rated by PTSD patients is predictive of treatment outcomes [e.g., (45, 46)], but Forbes et al. (47) and van Minnen et al. (48) reported no association between working alliance and outcome in their PTSD samples.

One potential reason for this discrepancy is methodological. In contrast to earlier studies, the cross-lagged analyses used in this study controlled for autocorrelations within each measure, which were high. The sample that provided patient alliance ratings was somewhat smaller than that for therapist ratings, restricting power. There was also some indication of restricted variance in patient alliance ratings in the later sessions and ceiling effects, and is in keeping with previous studies which have found that patient ratings of alliance tend to be fairly stable during treatment (1). Indeed, the patients' ratings of alliance in this study were consistently fairly high after all the three sessions 1, 3, and 5. It may be that their early first impressions of the therapeutic alliance, based on a first session of therapy that was engaging and collaborative, changed very little as treatment progressed and did not affect, nor was affected by, changes in their symptoms. Beck (49) wrote that a good therapeutic alliance is “necessary but not sufficient” to effect change in cognitive therapy. It may be that the “good enough” working alliance for most of the patients in this study was sufficient for engagement with treatment, but that the major influence on symptom change did not lie in their perception of the therapeutic relationship, but in the tasks and techniques used in treatment to produce cognitive change. This could suggest that therapists should prioritize establishing a solid working alliance in early sessions as a foundation for other aspects of treatment.

The reason for the discrepancy between the cross-lagged associations of PTSD symptoms of therapist and patient alliance ratings is unclear. Therapists do have more experience in the process of therapy than patients, and may be more likely to pick up on aspects of the alliance that will prove beneficial for future outcomes. Other studies, however, have found the opposite effect, with patients' ratings of alliance more predictive of outcome than therapists' [e.g., (1, 3, 50)]. Due to the methodological properties of the autoregressive, cross-lagged panel models (51) it cannot be ruled out that the alliance ratings partly reflected some trait-like stability. This might have led to the lagged parameters not only representing within-person relationships over time, but also between-person processes. This methodological problem may have been more pronounced for patients, some of whom had PTSD-related problems trusting other people in general which may have influenced their ratings. Indeed, a history of interpersonal trauma was related to lower initial ratings of the therapeutic alliance, which is in keeping with other studies that have suggested that people with a history of interpersonal trauma may particularly struggle to form a strong therapeutic alliance [e.g., (52)], but trauma type did not moderate the relationship between the working alliance and treatment outcome. However, the finding from the simple slopes analysis did indicate a potential effect of interpersonal trauma on the alliance-outcome relationship. This relationship requires further investigation.

Finally, the study aimed to explore the relationship between ruminative thinking and working alliance, following Brady et al.'s (18) finding that observer-rated ruminative thinking was associated with lower working alliance and predicted poorer outcomes in CT-PTSD. In this study, negative correlations between patient-rated ruminative thinking and therapist ratings of working alliance were found, but a non-significant positive correlation was found when patients rated the alliance. This indicates that therapists, but not patients, see rumination as an unhelpful strategy and thus rate alliance lower when this happens. The differential effect of ruminative thinking on patients' and therapists' rating may thus have contributed to the different pattern of results for the cross-lagged relationship with symptom reduction, as therapists are more effectively spotting that rumination is an unhelpful strategy, linked to poorer treatment outcome. Potential clinical implications of this finding are that therapists should address ruminative thinking in a manner which preserves the working alliance, such as collaboratively establishing the effect it has on the maintenance of PTSD symptoms.

A strength of this study was that it was drawn from a consecutive cohort of PTSD patients with a wide range of traumas and ethnic backgrounds who received an evidence-based psychological treatment in routine care and that the direction of the relationship between working alliance and symptom change during treatment could be investigated by repeated assessments.

Methodological limitations of the study include ceiling effects in the alliance measure that may have potentially masked effects, as a possible restriction in variance restricts magnitude of correlations and correlation-based parameters. The sample was of a similar size to other studies in this area, but would benefit from replication with a larger sample due to more complex analysis and estimation methods used in this study compared to previous studies. Another possible limitation is that the time lag between the therapy sessions was not always exactly 1 week, which may have led to some noise in the parameter estimates (53).

Despite these limitations, the study provides further insight into the relationship between working alliance and treatment outcome amongst patients receiving treatment for PTSD. It highlights the importance of a strong working alliance at the very start of treatment, possibly particularly with patients who have experienced interpersonal trauma and in addressing rumination. The mixed findings also indicate the importance of using ratings from multiple raters (therapist and patient) at multiple time points in treatment to fully understand the relationship between alliance and outcome in future studies.

## Data Availability Statement

The datasets presented in this article are not readily available because, we did not obtain consent to share patient data at the time of data collection but share the data analysis code and details of results. Requests to access the datasets should be directed to anke.ehlers@psy.ox.ac.uk.

## Ethics Statement

The studies involving human participants were reviewed and approved by King's College and South London and Maudsley Ethics Committee. The patients/participants provided their written informed consent to participate in this study.

## Author Contributions

EB wrote and revised manuscript, designed statistical analysis plan, and conducted statistical data analysis. HM wrote and revised manuscript, involved in literature review, contributed to treatment, data collection, and data analysis plan. MW involved in data management, contributed to data analysis plan, and critical revision of write-up. EW-P, JW, and RS contributed to treatment and clinical supervision, data collection, and critical revision of write-up. NG was the co-director of clinical treatment and clinical supervision, contributed to data collection, and critical revision of write-up. DC was the co-grant holder and contributed to critical revision of write-up. AE was the primary investigator of study and grant holder, designed and supervised data collection, clinical supervision, contributed to write-up, and critical revision. All authors contributed to the article and approved the submitted version.

## Conflict of Interest

The authors declare that the research was conducted in the absence of any commercial or financial relationships that could be construed as a potential conflict of interest.

## Footnotes

^1^Ehlers A, Wild J, Warnock-Parkes E, Stott R, Grey N, Cullen D, et al. Effectiveness of cognitive therapy in routine clinical care: Second phase implementation. (2021).
